# Development of an Approach of High Sensitive Chemiluminescent Assay for Cystatin C Using a Nanoparticle Carrier

**DOI:** 10.3389/fchem.2020.00802

**Published:** 2020-09-07

**Authors:** Yuanjie Sun, Liang Tao, Ying Ma, Shuya Yang, Xiyang Zhang, Boquan Jin, Zhujun Zhang, Kun Yang

**Affiliations:** ^1^Department of Immunology, The Fourth Military Medical University, Shaanxi, China; ^2^Key Laboratory of Analytical Chemistry for Life Science of Shaanxi Province, School of Chemistry and Chemical Engineering, Shaanxi Normal University, Shaanxi, China

**Keywords:** cystatin C, mesoporous silica nanoparticle, NaIO_4_ immobilization method, enhanced chemiluminescence, Rhodamine 6G, fluorescein

## Abstract

Cystatin C is an important cysteine protease inhibitor in the human body and is proposed as a new indicator of glomerular filtration rate for the detection of kidney damage. In this article, we report an ultra-sensitive, simple, and rapid chemiluminescence immunoassay method for cystatin C detection using functionalized mesoporous silica nanoparticles. After a three step hydrolysis, the amino-functionalized MSN encapsulating dye resulted in a hydrophobic environment for fixing the dye and amino groups for biological modification. The NaIO_4_ immobilization method maintained the activity of the antibody notably well. The sandwich immunoassay using two monoclonal antibodies was chosen for its selectivity. The analysis demonstrated that the detection upper was 0.0029 ng/mL and linear relationship within the range of 0.0035–0.5 ng/mL (*R*^2^ = 0.9936). The relative standard deviation (RSD) for 11 parallel measurements of 0.25 ng/mL CysC was 4.7%. The automated chemiluminescence analyzer could detect 96 wells continuously. The results demonstrated that this method is ultra-sensitive, simple, and rapid for detecting cystatin C.

## Introduction

Cystatin C (CysC) is considered to be an accurate marker of impaired renal function, especially in the early stages of Chronic Kidney Disease (CKD) (Mussap and Plebani, 2004). It is a nuclear protein that is freely filtered and almost completely degraded by tubular cells. Meanwhile, in the diagnosis and classification of CKD in diabetic patients, the CysC score is more sensitive than the Glomerular Filtration Rate (GFR) creatinine and GFR scores determined by the clearance rate of 51CR-EDTA, and early monitoring of renal failure is important in assessing the risk of CKD. In the prospective epidemiological study of myocardial infarction (PRIME), it is also considered a risk factor for cardiovascular disease (CVD) and is associated with coronary heart disease, and some researchers have found that CysC is associated with asymptomatic coronary artery disease (Beringer et al., 2009). Studies on brain diseases indicate that CysC plays an important role in various cerebrovascular diseases (Lee and Jung, 2017; Zhang and Sun, 2017; Zeng et al., 2019). Simultaneously, lung disease studies have shown that CysC levels have potential diagnostic value for the evaluation of respiratory function (Yoshizawa et al., 2015; El-Gammacy et al., 2018; Telo et al., 2018).

Various immunoassay methods for CysC have been reported. For example, heterogeneous immunoassay with radionuclide, enzymes and fluorophores labeling (Ma et al., 2010; Ristiniemi et al., 2012), particle enhanced turbidimetric immunoassay (PETIA) (Kyhse-Andersen et al., 1994), and particle enhanced nephelometric immunoassay (PENIA) (Finney et al., 1997). These immunoassay methods are highly sensitive and selective, and many can be detected more efficiently and quickly. However, some types of tagged immunoassay are not only expensive, but also cause environmental pollution.

Chemiluminescence detection is a new immunoassay technique. At present, more and more researchers have made breakthroughs in chemiluminescence detections research, and the application of chemiluminescence reactions has become more and more extensive. For example, It has been found a luminometer for in-line magnetic nanoparticle solid phase extraction and chemiluminescence measurement (Andrade et al., 2020). In addition, one chemiluminescence detection method was used ultrasensitive electro chemiluminescence aptasensor for detect kanamycin (Cheng et al., 2020). Zhong Yihong et al. also demonstrated that the CL imaging array method (Zhong et al., 2019). Zuo et al. (2018) have discovered chemiluminescent supramolecular nanoparticles used in anti-counterfeiting inks. Pilar et al. (2018) considered that an aqueous solution of gold nanoparticles can improve the efficiency in electrogenerated chemiluminescent reactions. Li et al. (2019) applied the imaging of local glucose levels in tumor periphery via peroxyoxalate chemiluminescent nanoparticle–glucose oxidase–doped alginate hydrogel.

In particular, Hui Lin team developed a label-free fluorescence and immune-independent detection method for CysC (Lin et al., 2013). Automated PETIA and PENIA can detect the susceptibility to *in vivo* indicators, such as hemolysis, blood lipids and bilirubinemia, etc., mainly because the light scattering signal may be affected by these factors. In addition, time-of-flight mass spectrometry (TOF-MS) is also used for CysC determination (Cotter et al., 2007), but the price of expensive instruments limits its wide application.

It is found that mesoporous silica nanoparticles (MSN) has potential value in the field of high sensitivity detection as carrier because of its good properties, such as semi-open nano-space, easy functionalization and high stability (Slowing et al., 2006; Cai et al., 2008; Zou et al., 2008). These properties allow a large amount of payload immobilized in the nano-channels, such as mediators, enzymes, and antibodies, to be involved in the reaction system (Lin et al., 2001; Slowing et al., 2007; Trewyn et al., 2007; Tsai et al., 2009; Vivero-Escoto et al., 2009; Zhao et al., 2009; Melde and Johnson, 2010). By loading various substances which could catalyze or be involved in chemiluminescence reactions, some chemiluminescence assay combined with MSN have a lower detection limit (Vinu et al., 2006; Lei et al., 2008; Roda et al., 2012). Nevertheless, the related research has still been not very sufficient.

Summary, the current extensive attention of researchers to CysC emphasizes the urgent need for rapid and reliable quantitative methods. In this article, we report an ultra-sensitive, simple, and rapid chemiluminescence immunoassay method for cystatin C detection using functionalized mesoporous silica nanoparticles (MSN).

We developed a new type of sandwich chemiluminescence immunodetection method for CysC labeled with an amino-functionalized MSN encapsulating dye. In the preparation stage, Methyl Trimethoxy Silane (MTMS) provides a hydrophobic environment in the nano-channels, the purpose is to fix the dye. At the same time, APTES is used to provide an amino group that fixes the monoclonal antibody to the surface of the nanoparticle. In the sandwich immunoassay process, CysC was measured based on the specific interaction between captured antigens and the anti-CysC monoclonal antibody labeled with functionalized mesoporous silica nanoparticles. First, in order to improve the utilization efficiency of dyes, the dyes were dissolved out of the MSN matrix using acetone in the chemiluminescence stage. Second, the chemiluminescence reagents were added using an auto-sampler. This method avoids the effect of mass transfer resistance and maximizes the utilization efficiency of dyes. The results showed that the method is sensitive, simple and rapid for the determination of CysC and is valuable for the determination of CysC in urine samples.

## Materials and Methods

### Reagents

Cystatin C, capture antibody and labeled antibody were provided by the FMMU (Fourth Military Medical University). Rhodamine 6G, Aminopropyl Triethoxysilane (APTES), Ethylene Glycol (EG), Tetraethoxysilane (TEOS), Fluorescein and Bis (2,4,6-trichlorophenyl), Cetyltrimethyl Ammonium Bromide (CTAB), Oxalate (TCPO), Methyl Trimethoxy Silane (MTMS) and other chemical reagents were purchased from Sangon Biotech (Shanghai, CHINA). The 96-well transparent microtiter plates were obtained from Corning Incorporated (Corning-Costar, Corning, NY).

The concentration of the coating solution is 0.05 mol/L carbonate buffer, pH 9.6. It contains 2.93 g NaHCO_3_ and 1.59 g Na_2_CO_3_ per liter. The PBS buffer (pH 7.4) was prepared by dissolving 8.0 g NaCl, 2.9 g Na_2_HPO_4_, 0.2 g KH_2_PO_4_, and 0.2 g KCl in 1 L of water. The 96-well plate was washed by PBST (PBS solution containing 0.05% Tween 20) solution. Cystatin C antigen and antibody were diluted with PBSB (PBS containing 0.1% BSA) solution as blocking solution. The PBS buffer (pH 7.1) was used to covalently immobilize monoclonal antibodies on nanoparticles.

### Preparation of the Amino-Functionalized MSN

This MSN formula is: (1) 40 mg of CTAB was dissolved in 50 mL of double-distilled water and stirred thoroughly until it is completely dissolved. Next, 2.1 mL of liquid ammonia and 570 μL of EG were dissolved in the CTAB solution, mixed well, and heated to 60°C. At <5 min, 200 μL of TEOS was added to the surfactant solution with a pipette, mixed well and let stand for half an hour. (2) 30 μL of MTMS, 2 mg of fluorescein and 20 mg of rhodamine 6G were added to the mixed solution, and mixed well. (3) After stirring the mixed solution at 60°C for 2.5 h with a magnetic stirrer, 80 μL of APTES was added to the previous reaction mixture. The stirring was continued at 60°C for 1 h. After filtration, a solid crude product was initially obtained, which was then washed with double distilled water. Then, ammonia solution, dye, CTAB surfactant and residual SiO_2_ precursor were removed by PBS dialysis method. Hence, MSN with rigidity, open macropore structure, and controlled pore size, this material can promote the mass transfer of fluorescent dyes and enable it to spontaneously capture signals in nano-channels.

### Covalent Immobilization of Monoclonal Antibodies Onto Nanoparticles

Oxidation of the antibody: (1) Anti-CysC monoclonal antibody (mAb) was dissolved in 70 μL acetate buffer (0.05 mol / L, pH 4.2). (2) 140 μL of NaIO_4_ solution (1.5 mg/mL, pH 4.2) was added to the above CysC antibody solution. (3) The sample was mixed well, and then let stand at 4°C in the dark for 2 h. (4) The reaction is terminated afterwards, the surface free NaIO_4_ is discarded by ultrafiltration tube centrifugation, and washed with acetate buffer multiple times.

Coupling of CysC mAb and nanoparticles: (1) 2 mg nanoparticles were washed 3 times with 5 mL PBS. (2) Then, the oxidized CysC mAb was added dropwise to the nanoparticle suspension, and mixed continually with the magnetic stirrer, and reacted at 4°C for 20 h. (3) The nanoparticles were centrifugally washed 3 times with PBS solution, and then resuspend with 600 μL PBS solution. (4) 20 μL of NaBH_4_ solution (2 mg/mL, pH 7.1) was added to the above solution, and then continuously stirred at 4°C for 2 h in the dark. (5) The obtained product was washed 3 times with PBS solution, and finally, resuspend in 500 μL of PBSB (PBS containing 0.1% BSA) and stored in 4°C.

### Sandwich Immunoassay

The process of sandwich immunoassay was as follows. (1) Coating, CysC capture mAbs were diluted with 0.05 mol pH 9.0–9.6 carbonate buffer to a working concentration of 10 μg/mL, 100 μL working solution was added to each polystyrene plate reaction well, and incubate at 4°C for 18–24 h. The next day, the solution in the well was discarded and washed 3 times with PBST solution for 3 min each time (referred to as washing, the same below). (2) A certain concentration CysC standard was added to the above coated wells, which was incubated at 37°C for 2 h and then washed. (3) 100 μL the MSN detection antibody complex freshly diluted with PBSB were added to each reaction well, and incubated at 37°C for 90 min and washed. (4) The substrate solution was added to develop color, and 100 μL of temporarily prepared TMB substrate solution was added to each reaction well and incubate at 37°C for 10–30 min. (5) 50 μL of 2M sulfuric acid was added to each reaction well to terminate the reaction. (6) To judge the result, read the value and draw a graph.

### Chemiluminescence Assay

The chemiluminescence assay was accomplished by introducing TCPO-H_2_O_2_-imidazole-fluorescent dye chemiluminescence reaction system. The imidazole, hydrogen peroxide and TCPO were diluted with acetonitrile solution (containing 30% acetone). The CL detection was carried out by Synergy^TM^2 SLD instrument of Bio Tec Corporation in the United States.

The chemiluminescence assay was conducted as follows: (1) After the sandwich immunoassay, 50 μL of acetone was added and continuously shook for 5 min. (2) The 96-well plates were kept at 37°C until the acetone volatilized completely. (3) Then, the 96-well plates was placed into the microarray scanner Synergy^TM^2 Multi-Mode Microplate Reader (Bio Tec instruments, Inc., www.biotek.com). 50 μL of hydrogen peroxide-imidazole solution and 50 μL of TCPO solution (3.0 × 10^−4^ mol / L) were added to the 96-well plates by automatic sampler attached to the instrument. At the same, the signals were acquired for the 96-well plate.

### Urine Sample Treatment Methods

The urine sample was centrifuged at 16,000 rpm for 15 min at room temperature, then the supernatant was filtered through filter paper and collected into a centrifuge tube. Different concentrations of CysC was added to the urine, and diluted into equal volumes of samples containing different concentrations of CysC to be detected.

Above, the implementation of this experiment was approved by the FMMU Ethics Committee. All patients participating in the experiment understood all the details of the experiment and agreed to implement it.

## Results and Discussion

### Characterization of Amino-Functionalized MSN

**Figure 1:**
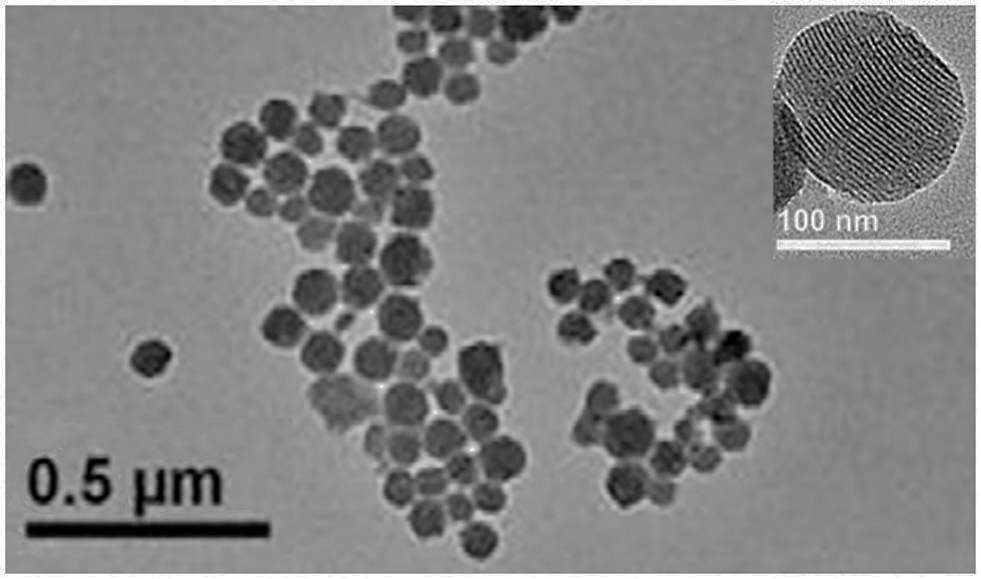
TEM images of the MSN.

The Figure 1 clearly showed that the morphology of MSN was mesoporous materials. In the present study, the numbers of hydrophobic groups on MSN supplied by MTMS were used to separate dye from water phase (Cho et al., 2010). However, its application was limited due to lack of functional groups. The generation of amino groups through the hydrolysis of APTES has provided with biological convenience. The introduction of amino groups improved the dispersion of MSN in water and made it easy to mark. The previous study shows that increasing the APTES concentration, gradually changes the pH of the nanoparticle dispersion to alkaline. The optimized dosage of APTES was 0.08 mL in 50 mL of synthetic system (Tao et al., 2013).

Rhodamine 6G and Fluorescein must be encapsulated in MSN at the same time. In the experiment, we found that the production of dispersed MSN singly encapsulated Rhodamine 6G were much less than which encapsulated two dyes. The reason of this phenomenon needs to be studied further. One of the possible explanations was that electropositivity of Rhodamine 6G cause electrostatic agglomeration in reaction system. When two dyes are added in the system at the same time, the positive of Rhodamine 6G has been neutralized by the negative of fluorescein. The dispersibility of nanoparticles affected the efficiency of the labeling reaction, which determined the quality of bio-functionalizated MSN. The encapsulating double dyes resolved this problem well and increased the loading amount of dye at the same time.

### Characterization of Assay

**Figure 2:**
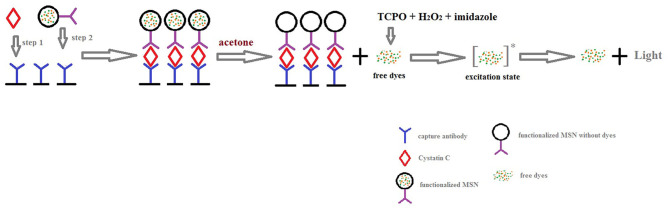
Diagrammatic sketch of chemiluminescence sandwich immunoassay of CysC.

The diagrammatic sketch of our method is shown in Figure 2. Compared with other immunoassay methods, the sandwich immunoassay selects two paired monoclonal antibodies as capture antibody and detection antibody respectively. It is considered to be the best method in specificity. Thus, sandwich immunoassay was recognized as the final immunoassay model.

In Figure 2, the oxalic acid peroxide ester chemiluminescence reaction mechanism is also exhibited. First, oxalate nucleophilic attack of the carbonyl generated produces the high energy double oxy cyclic intermediate dioxin butanedione. Second, the intermediate decomposition energy is transferred to the acceptor fluorescent molecules in the excited state. Finally, the excited molecule returns to the ground state from the excited singlet state, releasing a photon that is emitted as fluorescence. Because it has up to 30% of the chemiluminescence quantum yield and is also a steady chemiluminescence, the peroxide oxalate chemiluminescence reaction system has a higher sensitivity than other chemiluminescence systems.

**Figure 3:**
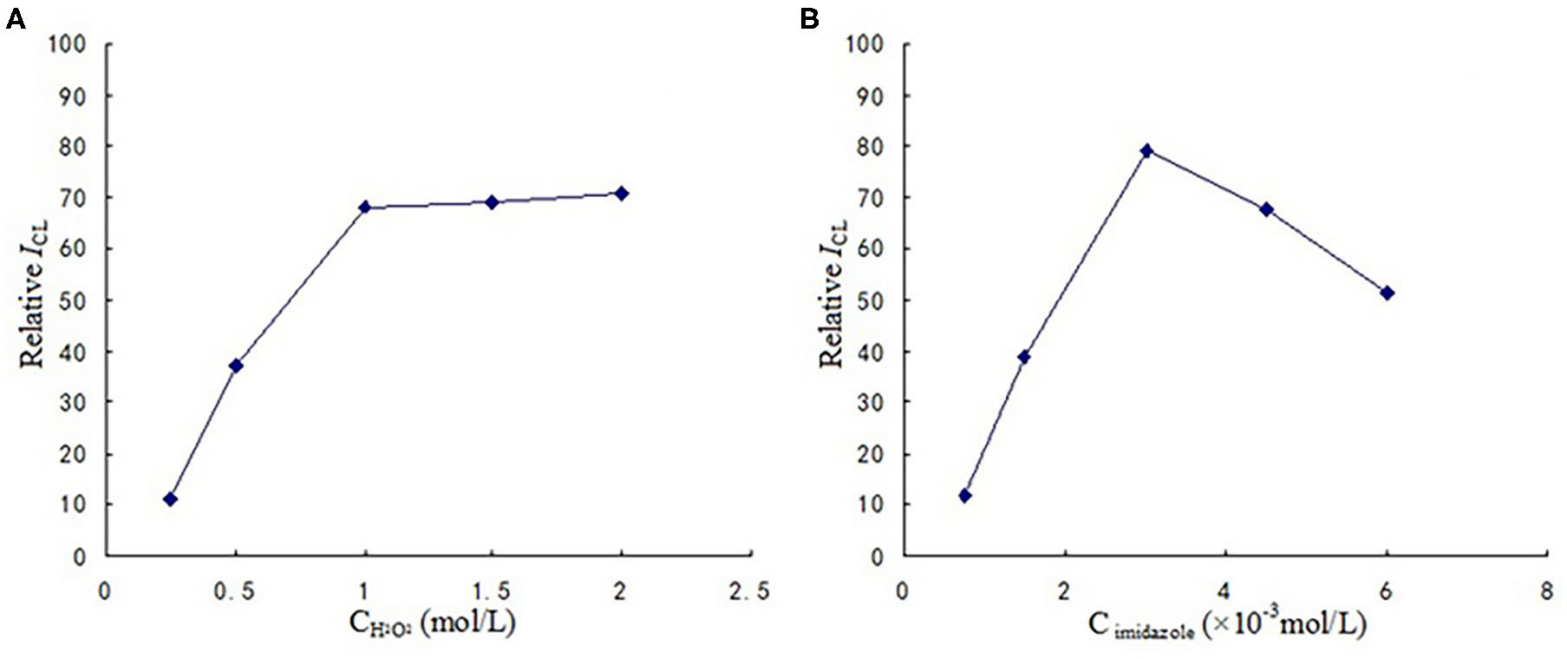
**(A)** The optimization of H_2_O_2_ concentration. **(B)** The optimization of imidazole concentration.

The optimized reaction conditions are shown in Figure 3. It can be observed that concentration of hydrogen peroxide and imidazole were 1 mol/L, 3 × 10^−3^ mol/L respectively.

An important development in this method is the addition of a step of dissolving the dyes in the nano-matrix with acetone prior to the chemiluminescence reaction. Because the mesoporous structure of MSN is semi-open structure, the mass transfer resistance of this structure has some influence on the chemiluminescence reaction. This point makes the chemiluminescence reaction take place on the surface of the material. The improvement released deep dyes and improved the utilization efficiency of dyes in nano-matrix, thereby ultimately achieving the purpose of reducing the detection limit.

**Figure 4:**
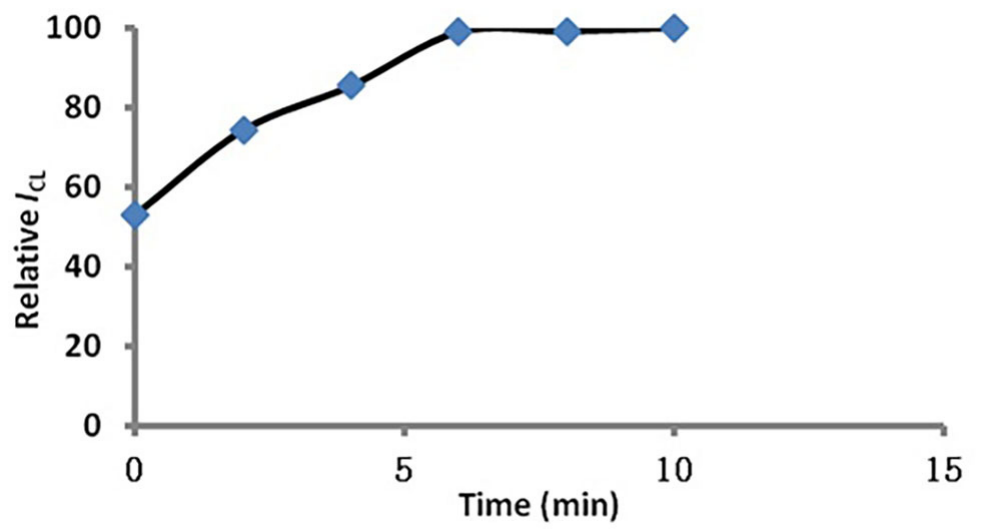
Influence of time on dye dissolution.

In order to ensure that MSN within the dyes can be completely stripped, the effect of time for dye dissolution was tested. From Figure 4 we can see that the dyes were completely dissolved by acetone after 6 min. So we used 6 min as the optimized condition.

### Optimization of the MSN-Labeled Sandwich CL Immunoassay

**Figure 5:**
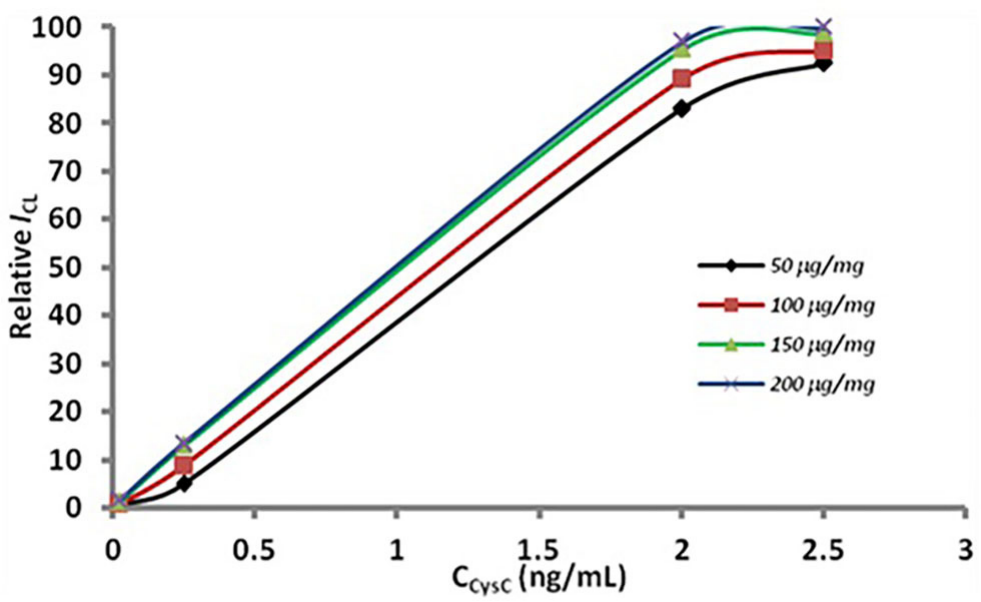
Optimization of antibody dosage. The concentration of the antibody in modification were 50 (the rhombus), 100 (the box), 150 (the triangle), 200 (the fork) μg per mg MSN.

In the sandwich immunoassay the conditions of the two methods were identical. The results have been show in Figure 5. It can be observed that the CL intensity obviously increased, as the amount of antibody on the nanoparticle surface increased from 50 to 150 μg. When the antibody dosage exceeded 150 μg per mg MSN, the CL intensity increased slowly. Thus, the optimum antibody density is 150 μg per mg MSN considering CL intensity and reagent consumption.

**Figure 6:**
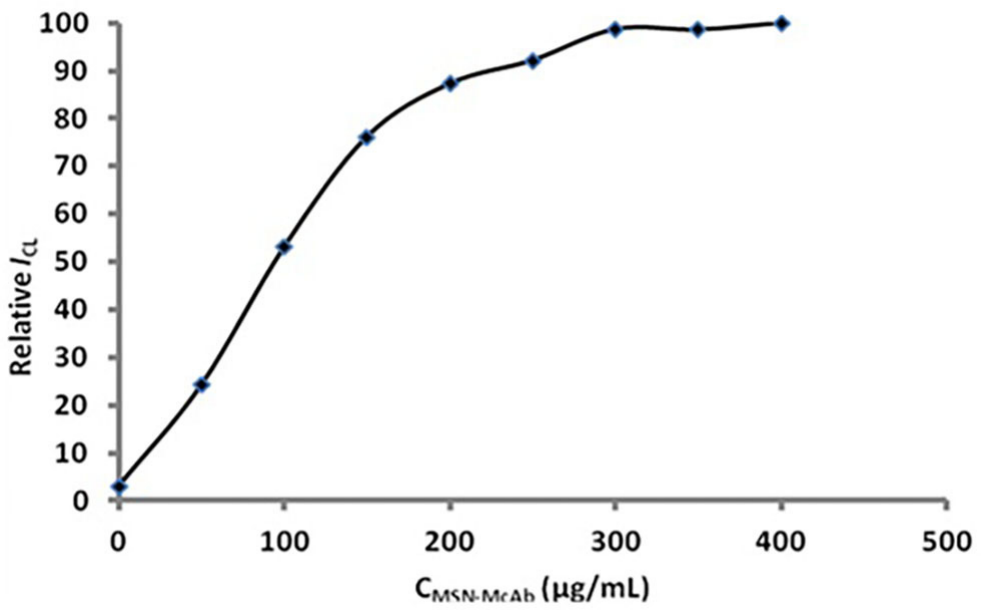
Optimization of the concentration of MSN-antibody.

The concentration of the MSN-antibody solution also played an important role in the immunoassay (Figure 6). From Figure 6, we can see that the signal obviously increased when the concentration of the MSN-antibody was lower than 300 μg/mL. When the concentration is higher than 300 μg/mL, the CL intensity will increase slowly due to the near-saturated non-specific adsorption. It can be seen that the optimal concentration of the MSN antibody solution is 300 μg/mL.

### Detection of CysC

**Figure 7:**
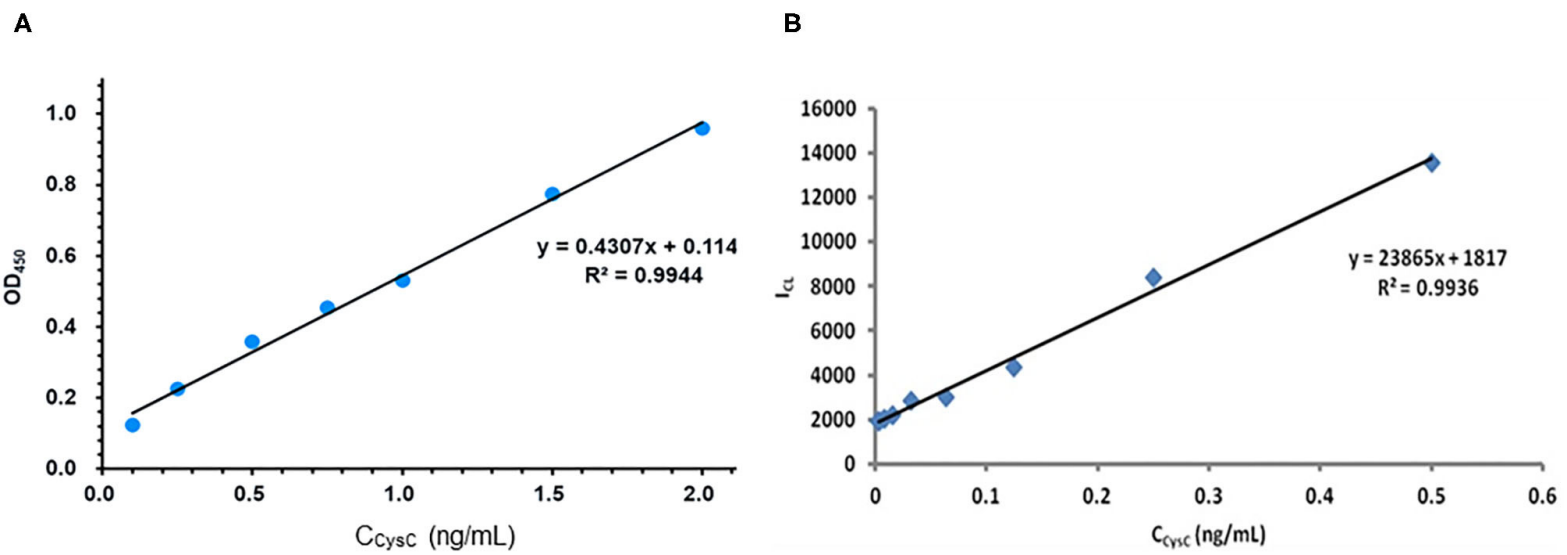
Compared with the performance of ELISA method and the sandwich chemiluminescence immunoassay using MSN as label. **(A)** The ELISA method. **(B)** The sandwich chemiluminescence immunoassay.

The comparisons of two methods' performances are exhibited in Figures 7A,B and Table 1. As shown in Figure 7A the ELISA method of the linear range was 0.1–2 ng/mL, and the detection limit was 0.033 ng/mL (3σ). And in Figure 7B, the linear range of the dye stripping method was 0.0035–0.5 ng/mL. The detection limit was 0.0029 ng/mL (3σ). Compared with the ELISA method, the proposed method demonstrates considerably more sensitive than the direct method (Table 1). Normally, an antibody molecule can be labeled with about ten dye molecules. When MSN is used as marker, a lot of dye molecules can be labeled on of antibody. This development greatly improves the sensitivity of the method. The reason is that mesoporous silica nanoparticle matrix is a semi open carrier, which can be loaded with a large number of dye molecules.

**Table 1:** Comparison between ELISA and CL method.

	**ELISA**	**CL method**
R^2^	0.9944	0.9936
Linear range	0.1–2 (ng/mL)	0.0035–0.5 (ng/mL)
RSD (%)	4.6% (*n* = 11)	4.7% (*n* = 11)
LOD	0.0333 (ng/mL)	0.0029 (ng/mL)

### Determination of CysC in Human Urine Samples

After heart surgery in adults, acute kidney injury may accompany it. The content of CysC in urine can be used as a marker for early diagnosis of renal function, which is better than conventional plasma markers. This study can effectively assess the applicability and accuracy of this experimental research method by detecting five different concentration gradients of CysC in the urine samples of patients. The results of the sample analysis are shown in Table 2. It can be observed that the two methods compared with ELISA show no significant difference in the detection range.

**Table 2:** Analytical results of CysC in samples.

	**ELISA (ng/mL)**	**CL method (ng/mL)**	**Difference (%)**
Urine 1	21.34	22.115	4.1
Urine 2	54.76	51.202	−6.5
Urine 3	18.58	20.196	8.7
Urine 4	2.36	2.485	5.3
Urine 5	7.64	7.096	−7.1

All results of this study are based on at least three independent experiments.

## Conclusions

In our current research, a novel ultra-sensitive, simple, and rapid chemiluminescence immunoassay method for detecting CysC was developed using an amino-functionalized MSN encapsulating dye as a label. According to its semi-open advantage, the obtained MSN can release the dye for maximum efficiency in chemiluminescence immunoassays. Compared with many methods for detecting CysC, the proposed chemiluminescence immunoassay method shows better performance in detection limit.

## Data Availability Statement

The raw data supporting the conclusions of this article will be made available by the authors, without undue reservation.

## Author Contributions

This study was completed by Prof. Yang's group and Prof. Zhang's group. They guided this research. YS established the auto flow injection chemiluminescence array assay, wrote the main manuscript text and prepared figures. LT established the ELISA method of Cystatin C, and also completed the separation and purification of the antibody. The hybridoma cell was screened by YM. SY and XZ were doctor students and participated in all the experiments under YS's guidance. Cycs monoclonal antibodies belonged to BJ and were provided by BJ. All authors reviewed the manuscript.

## Conflict of Interest

The authors declare that the research was conducted in the absence of any commercial or financial relationships that could be construed as a potential conflict of interest.
